# Recent applications of boxed molecular dynamics: a simple multiscale technique for atomistic simulations

**DOI:** 10.1098/rsta.2013.0384

**Published:** 2014-08-06

**Authors:** Jonathan Booth, Saulo Vazquez, Emilio Martinez-Nunez, Alison Marks, Jeff Rodgers, David R. Glowacki, Dmitrii V. Shalashilin

**Affiliations:** 1 School of Chemistry, University of Leeds, Leeds LS2 9JT, UK; 2 Departamento de Química Física and Centro Singular de Investigación en Química Biológica y Materiales Moleculares, Campus Vida, Universidad de Santiago de Compostela, 15782 Santiago de Compostela, Spain; 3 School of Life Sciences, University of Bradford, Bradford BD7 1DP, UK; 4 School of Chemistry, University of Bristol, Bristol BS8 1TS, UK; 5 Department of Chemistry, Stanford University, Stanford, CA 94305, USA

**Keywords:** molecular dynamics, quantum mechanics, dynamics of proteins, chemical dynamics, kinetics

## Abstract

In this paper, we briefly review the boxed molecular dynamics (BXD) method which allows analysis of thermodynamics and kinetics in complicated molecular systems. BXD is a multiscale technique, in which thermodynamics and long-time dynamics are recovered from a set of short-time simulations. In this paper, we review previous applications of BXD to peptide cyclization, solution phase organic reaction dynamics and desorption of ions from self-assembled monolayers (SAMs). We also report preliminary results of simulations of diamond etching mechanisms and protein unfolding in atomic force microscopy experiments. The latter demonstrate a correlation between the protein's structural motifs and its potential of mean force. Simulations of these processes by standard molecular dynamics (MD) is typically not possible, because the experimental time scales are very long. However, BXD yields well-converged and physically meaningful results. Compared with other methods of accelerated MD, our BXD approach is very simple; it is easy to implement, and it provides an integrated approach for simultaneously obtaining both thermodynamics and kinetics. It also provides a strategy for obtaining statistically meaningful dynamical results in regions of configuration space that standard MD approaches would visit only very rarely.

## 1. Introduction

Boxed molecular dynamics (BXD) [1–3] is a simple and straightforward technique that extends the time scale of atomistic molecular dynamics (MD) simulations and facilitates simulation of rare events. In BXD's simplest implementation, we assume that a chemical reaction or some other atomistic physical process can be described by some reduced description of the system's 3*N* dimensional configuration space—e.g. a reaction coordinate or an appropriate order parameter. It is then possible to split this coordinate into several boxes and subsequently lock the dynamics in each box by inverting the velocity of the trajectory in the direction of the reaction coordinate (or order parameter) every time the trajectory hits a boundary between two subsequent boxes. Then, one can calculate the rate constant of exchange between the boxes simply by calculating average time between the ‘hits’.
1.1
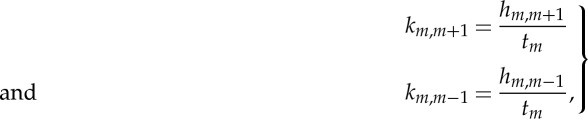

where *h*_*m*,*m*−1_ and *h*_*m*,*m*+1_ are the number of ‘hits’ of the left and right boundary of the *m*th box, and *t*_*m*_ is the time spent in this box. After accumulating sufficient statistics, the trajectory is allowed into the neighbouring box where the procedure is repeated. Eventually, a set of rate constants for exchange between the boxes is accumulated, and the dynamics is reduced to a set of kinetic equations.
1.2
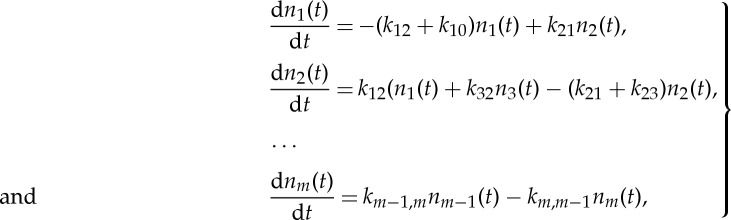

or in the matrix form
1.3
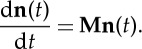

Then, the MD simulation of the system is replaced by the solution of the master equation (1.2)
1.4
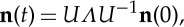

where ***n***(0) contains the initial conditions for each box, *U* is the eigenvector matrix obtained from diagonalization of **M**, and *Λ* is a diagonal matrix whose elements, *Λ*_*jj*_=e^λ_*j*_*t*^, are determined by **λ**, the eigenvalue vector corresponding to **M**.

BXD relies on the assumption that the motion within the box is stochastic and that sequential ‘hits’ and velocity inversions are uncorrelated—i.e. the time between the ‘hits’ must be bigger than the correlation time. In general, this is not the case. A trajectory reflected from a boundary can sometimes turn back rather quickly. One needs to use a simple technique described in [2] in order to remove these short-time-correlated events. The requirement of uncorrelated dynamics also imposes a restriction on the box size—i.e. it should be bigger than the so-called correlation length, which is the length at which a trajectory loses the memory of its initial conditions. For large anharmonically coupled systems, the correlation time and correlation length are usually quite short. The procedure developed in [2] is based on analysis of the distribution of first passage times. It removes any contribution to the rate coefficients from velocity inversions separated by very short time and improves the quality of the kinetic results.

With the box-to-box rate coefficients in hand, it is possible to estimate the free energy profile along the reaction coordinate:
1.5
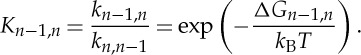

**Figure 1.**
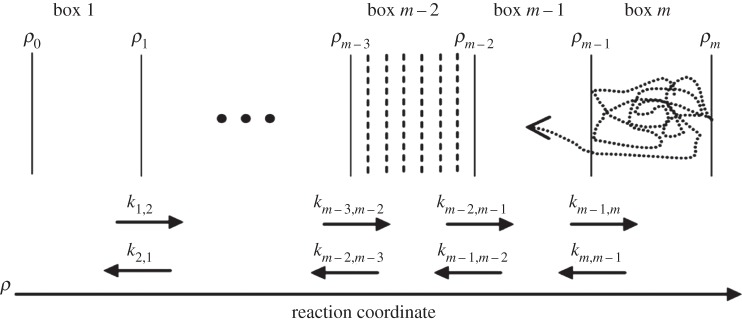
Schematic of how BXD works with a reaction coordinate, *ρ*, split into *m* boxes.

A quantity which is closely related to the free energy is the potential of mean force (PMF). Using BXD, it has been shown [1] that the free energy (or PMF) profile can actually be estimated at a resolution much higher than the box size. The procedure involves splitting boxes into smaller regions and calculating the time spent within those smaller regions (enclosed by dashed lines in figure 1). This gives a high-resolution free energy profile within each box. These profiles can then be stitched together using an exact procedure that yields the global free energy profile along the entire reaction coordinate. We have also developed a post-analysis procedure for calculating kinetic rate coefficients between small boxes in the cases where that information is useful [3].

For us, BXD has its origin in the intramolecular dynamics diffusion theory (IDDT) [4–8], which has demonstrated that long-time reaction rates can be recovered from a set of short-time MD simulations [8]. Longer time-scale dynamics may be calculated from the set of rate constants obtained from short-time simulations within each box. This connection between short and long time scales is what makes BXD a multiscale method. In the IDDT, the dynamics was not restricted by the boundaries of the box. The idea of the box was introduced in [9], where the first BXD simulation of bond fission was carried out using only two boxes with a single boundary between them. Locking the trajectory within a box enables the system to visit the regions of high free energy and low probability, where an unrestricted trajectory would rarely visit.

Rare event acceleration is an important part of modern MD simulations. Over the past few years, a number of rare event acceleration strategies have been proposed, many of which share similarities with BXD. Milestoning [10–15] and particularly milestoning with Voronoi tessellations (MVT) [13,16] are the most closely related to the BXD technique, and it is worthwhile discussing connections between BXD and milestoning. Both use short-time dynamics to recover local kinetic information about the motion along the reaction coordinate in order to solve a kinetic state-to-state master equation. The original milestoning procedure required accurate sampling of the equilibrium distribution at the milestone surfaces located along the reaction coordinate. This is very similar to the earlier IDDT procedure [7,8], with the primary difference in the post-processing step: milestoning analysis relies on the milestone-to-milestone passage time, and a IDDT analysis relies on a diffusional coefficient. In MVT [13,16], a modified milestoning procedure has been proposed wherein the dynamics initiated between a set of milestones are then locked using a collision rule at the boundary so that they cannot escape, as in [9]. Perhaps the most important difference between the velocity inversion scheme used by BXD and the collision rule of MVT is that the BXD scheme conserves energy, linear momentum and angular momentum. So long as the distance between a BXD inversion boundary and transition state is larger than the characteristic system decorrelation length, then the accelerated dynamics obtained using BXD give meaningful statistics that allow detailed analysis of non-equilibrium dynamical properties in the post-transition state region of a given molecular system's phase space (see [2]). Indeed, these features of BXD have proven critical to many of the studies which are outlined in this paper, where BXD was used to investigate non-equilibrium energy relaxation in number of particles, volume, energy (NVE) microcanonical ensemble trajectories [17–19].

BXD and MVT also differ in the post-processing step—i.e. how they interpret the MD simulation results obtained from within the boxes. BXD uses the trajectory information to calculate box-to-box transition state theory rate constants and MVT uses the information to calculate transition times for moving from border to border. As has been shown in [2] calculating either of the two is not entirely straightforward and requires thinking about dynamical decorrelation. In BXD, the decay trace needs to be corrected by removing fast events where trajectories return to the boundary. This gives decay traces which are better approximated by a single exponent and box-to-box dynamics better described in terms of a single rate constant. MVT, on the other hand, gives flat non-exponential decays at short times. When these are removed, then the BXD and MVT decay traces are identical. The foundation for MVT is based on ideas from transition path sampling, and it is exact in the limit that the correct set of milestones is used [14]. BXD has its foundations in standard transition state theory. It is exact in the limit that the system is ergodic, and a correct set of dividing surfaces has been used. Based on BXD's origins in transition state theory, we have shown how to simply and exactly renormalize the BXD statistics to obtain thermodynamics [1] and kinetics [3] to arbitrary histogram resolution, distinct from milestoning and MVT.

BXD also shares similarities with a number of other rare event acceleration methods. For example, BXD in its simplest form [9] is related to hyperdynamics [20,21], which similarly introduces constraints into a molecular system's configuration space to encourage it to visit regions of low probability. BXD also has similarities to umbrella sampling [22–25] with the primary difference that the former uses rectangular boxes instead of parabolic force restraints. Compared with these methods, BXD's main advantage is that it is the only technique which is capable of simultaneously extracting high-resolution thermodynamic and kinetic information. BXD's formulation is very straightforward, based upon a simple rewriting of the classical transition state theory formula, which is familiar in chemistry. In common with all of the aforementioned techniques, BXD does not address the question of finding a good reaction coordinate or order parameter; its usage relies on being able to define these in a sensible way. In many instances, definition of an appropriate reaction coordinate or order parameter is a substantial challenge, but there are a range of systems where these quantities are reasonably well defined. If a good reaction coordinate exists, then BXD provides long time-scale dynamics/kinetics and PMF profiles in regions of very high energies.

In this paper, we review several recent and ongoing applications of the BXD technique. First, we review the simulation of peptide loop formation dynamics where interesting and unusual power law kinetics observed in experiments [26–28] have been reproduced on time scales spanning several orders of magnitude—i.e. from picoseconds to microseconds. Second, we report new results from our simulations of ion diffusion through a monolayer on a metal surface, where residence times of hours were recovered in agreement with experiment [29–31]. Third, we give a brief summary of how BXD has been used to provide insights into the elementary chemical mechanisms of diamond etching. Fourth, we describe how BXD has been used to accelerate rare event sampling in studies of solution phase bimolecular reactions. Finally, we report preliminary BXD results of the free energies obtained from pulling apart several small proteins, all of which were previously investigated experimentally using atomic force microscopy (AFM) [32–34]. For these systems, we observe that the slope of the preliminary PMFs calculated as a function of peptide extension (distance between the peptide's C and N termini) appear to be in reasonable agreement with the experimentally observed AFM forces required to unfold the proteins. For proteins composed of *α*-helices, the force required along the extension coordinate is substantially lower than for those composed of β-sheets, in qualitative agreement with experimental observations. We also discuss future developments and applications.

## 2. Applications of boxed molecular dynamics

### (a) Peptide loop formation

**Figure 2.**
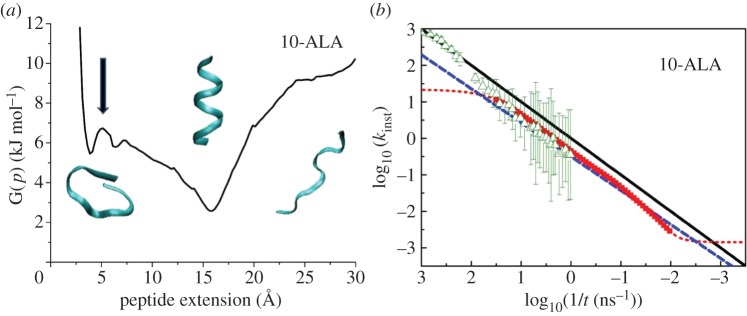
BXD results obtained from 10-ALA. (*a*) The PMF as a function of extension coordinate. The arrow indicates the transition state for going from the extended conformation to the closed loop form. (*b*) The results of BXD simulation (thick red line) together with the outcome of unbiased short-time MD simulations (triangles). The two plateaus shown by the red dashed line correspond to the smallest and largest eigenvalues of the matrix **M** in equation (1.3) and therefore represent the limits between which the BXD master equation results are valid. The result is also compared with a 1/*t*^*α*^ power law, with *A*=1, *α*=1 (black line) and *A*=3.2,*α*=0.93 (blue dashed line). (Online version in colour.)

Volk and Hochstrasser previously carried out a series of detailed studies investigating the kinetics of polypeptide cyclization. Using a laser pulse, their experiments photolytically cut a solvated polypeptide loop (in which the two termini of the polypeptide backbone were connected via a disulfide bond), generating a thiyl radical pair localized on each end of the peptide [26–28]. The radical concentration was monitored by time-resolved spectroscopy spanning picoseconds to milliseconds [27,28]. The measured absorbance of the peptide occurs predominantly from the thiyl radicals (a configuration denoted below as *n*_open_), decreasing when the radical pairs recombine to form the bond between the two ends of the polypeptide chain, i.e. when the peptide loop is reformed. In these experiments, the instantaneous rate constant [26–28]
2.1
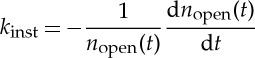

was determined to have the form
2.2
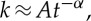

where *k* has units of ns^−1^, *A*=3.2 and *α*=−0.93±0.02, with similar values observed for loop formation dynamics in a range of peptides. Remarkably, this time-dependence, which is very close to simple *k*=1/*t* dependence, holds for nine orders of magnitude in time, from picoseconds to milliseconds, and was essentially the same for a short polypeptide [27] and a 100-residue protein [28]. To rationalize these experimental results, we performed the first fully atomistic simulations of the system, using BXD to investigate several model peptides with initial conditions corresponding to those of experiment, i.e. at *t*=0 the two ends of the peptide are in close contact. In our simulations, the reaction coordinate was taken to be the peptide extension. This coordinate was split into anywhere between 15 and 20 boxes, with sizes ranging from 1 to 2 Å. Box-to-box rate constants were obtained using a decorrelation algorithm which we have recently described [2]. The procedure developed in [3] was used to extract ‘high-resolution’ rate coefficients from the low resolution box-to-box rate coefficients. Having obtained the high-resolution rate coefficients, a solution (1.4) of the kinetic master equation (1.3) was obtained. Figure 2 shows BXD results obtained for one of the peptides investigated in this study, namely 10-ALA.

Frame (*a*) depicts the PMF as a function of the reaction coordinate. Frame (*b*) presents the instantaneous rate constant of cyclization of the 10-ALA peptide. Triangles show the rate constant obtained by unbiased MD simulation. Using a serial implementation of the CHARMM program suite [35] on a local computing cluster, these unbiased simulations only permit reaction events on a picosecond time scale. The red line demonstrates the outcome of the BXD simulation. The plateaus shown by the red dotted line indicate the limitations of BXD—i.e. the BXD results are only reliable for times *t*, where *t*_max_>*t*>*t*_min_, where *t*_max_ and *t*_min_ are the longest and shortest time scales obtained from the eigenvalue spectrum, respectively. As can be seen from figure 2, BXD reproduces a power law very similar to those obtained from fitting of the experimental data, which are shown by blue and black lines. Using BXD, atomistic simulations can be used to derive kinetic information on the microsecond time scale, far beyond time scales which are accessible by unbiased MD.

Similar results were obtained for a number of model peptides and it was shown that for all of them the instantaneous rate closely follows the power law (2.2). The behaviour appears to be largely independent of the structure of peptides and it can be explained on the basis of the solution (1.4) of the master equation (1.3), giving the following form for the time-dependence of the extended peptide
2.3
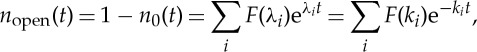

where *n*_0_(*t*) is the population of the box 0, which corresponds to loop closure, and *F*(λ_*i*_) is a pre-exponential factor that weights the contribution of a particular eigenvalue to the overall time-dependent population of extended peptide, *n*_open_(*t*). The rate constants *k*_*i*_=−λ_*i*_ of the exponents in (1.4) are related to the eigenvalues of the kinetic matrix **M** in (1.3). It was noted in [3] that the coefficients *F*(λ_*i*_) depend strongly on the initial populations of the boxes *n*_*i*_(0). Our analysis focused on the eigenvalue spectrum weighted by the corresponding coefficients *F*(λ_*i*_). The shape of this weighted spectrum determines whether the phenomenological kinetics follow a power law or a standard form of a single exponent. In the limit that a single value in the weighted spectrum dominates, typical single exponential kinetics are observed. In the limit that there are no dominant values in the weighted spectrum, power law kinetics arise. In the immediate aftermath of experimental bond-breaking, all the population is concentrated in the ‘boxes’ adjacent to the loop formation transition state. This results in a scenario where the weighted spectrum has no dominant values, and all the rate coefficients *k*_*i*_ have characteristic times *t*_*i*_=1/*k*_*i*_, that are equally probable, giving rise to the time-dependence (2.2) for the instantaneous rate (2.1).

**Figure 3.**
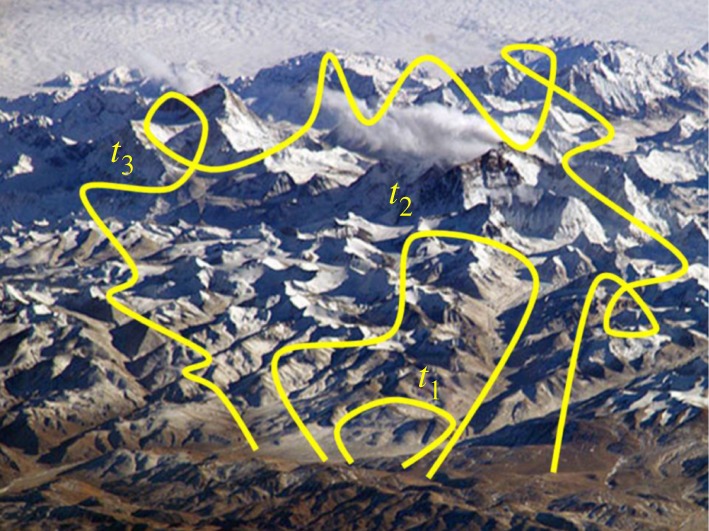
Routes selected randomly on a landscape. At time *t*_2_, the hikers who took route *t*_3_ are still walking but the hikers on the short route *t*_1_ have already returned. Therefore, at the moment *t*_2_, the instantaneous rate constant of return is equal to 1/*t*_2_. If the start and endpoints of all routes are in close proximity, then there is a large number of short routes, which is not the case when start and endpoint are distant from each other. (Online version in colour.)

These results can be understood with the help of a simple non-scientific analogy. Suppose that a large group of hikers randomly choose their routes through a landscape similar to that shown in figure 3, which provides a schematic representation of the energy landscape of a peptide. The randomness of each hikers' path means that there will be a distribution of available paths, each of which has a unique characteristic time for returning home. At time *t*_2_, for example, those hikers who took a route with characteristic time *t*_1_ have already returned, whereas those on a longer route *t*_3_ are still far away. Thus, at a given time *t*, only the routes with the rate constant close to *k*=1/*t* contribute, which explains the origin of the 1/*t* law in (2.3). An important feature of the scheme depicted in figure 3, which distinguishes the ‘returning hiker’ analogy from numerous existing random walk models, is that the all routes are initialized and terminated in roughly the same place. This means that shorter routes contribute similarly to longer routes.

The example of peptide cyclization not only demonstrates that BXD can produce reliable thermodynamic and kinetic information on very long time scales (allowing one to reproduce experiments), but that it also helps to provide simple insights into the qualitative mechanisms that give rise to the experimental observations.

### (b) Desorption of ions from the monolayer

**Figure 4.**
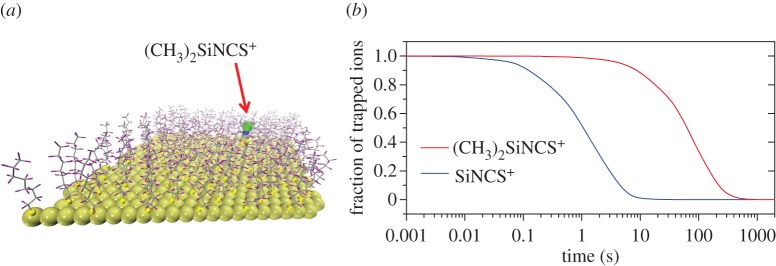
(*a*) Monolayer of perfluorinated alkanethiol on gold with adsorbed ion; (*b*) survival probability of SiNCS^+^(red) and (CH_3_)_2_SiNCS^+^(blue) in the monolayer calculated as a fraction of the trapped molecules. (Online version in colour.)

As has been shown in the previous example, BXD is capable of reproducing the dynamics of peptides on time scales of microseconds where the barriers along the PMF are relatively small. An important additional question is whether BXD can accurately treat processes with much larger energy barriers along the PMF, where the dynamical time scales are substantially slower. In a recent study [36], the dynamics of the desorption of silyl ions (CH_3_)_2_SiNCS^+^ and SiNCS^+^, trapped in the self-assembled monolayer of perfluorinated alkanethiol on gold (F-SAM) has been investigated. The monolayer with (CH_3_)_2_SiNCS^+^ adsorbed on it is shown in figure 4*a*. In the experiment [29–31], a monolayer was bombarded with each of these ions (separately) during about 1 h, forming ions adsorbed just beneath the surface of the monolayer (figure 4*a*). Subsequently, the F-SAMs were analysed by high-energy collisions of Xe^+^ ions, which induce the ejection of surface species (sputtering). The sputtered ions were detected by mass spectrometry. It was shown that the larger ion (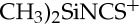 was observed in the mass spectrum after several days, in stark contrast to the complete lack of the smaller SiNCS^+^ ion. The interpretation given in the experimental studies is that the methyl groups increase the attractive and steric interactions between the ion and the F-SAM, which facilitate the entrapment of the ion inside the monolayer and decrease its desorption rate.

To address whether atomistic MD simulations were able to provide any insights into these experimental observations, we carried out BXD simulation of the ions diffusing inside of the monolayer towards the surface with subsequent desorption. For a reaction coordinate, we used the distance from the ion centre of mass to the surface. The residence time was estimated using the kinetic master equation. The BXD module in the CHARMM code was used, and BXD box-to-box rate constants were obtained. The rate constants were corrected by the previously discussed decorrelation procedure, which excludes the correlated velocity inversions separated by very short time. The rate constants included an additional correction, designed to account for the presence of image charge at the gold surface. This procedure allows one to compute the survival probability for ions diffusing from the monolayer to the vacuum, which is shown at figure 4*a*, yielding the residence time of 91 s for (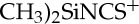 and 1.5 s for the more compact SiNCS^+^ ion, which explains why SiNCS^+^ is not seen in the mass spectrum at long times.

This system provides a nice demonstration of the fact that BXD is capable of reaching time scales which are many orders of magnitude longer than the time scales typically recoverable by atomistic simulations. This is because of BXD's multiscale approach: atomistic simulations yield box-to-box rate constants, which are used as subsequent input for kinetic analysis, covering much longer times.

### (c) Heterogeneous chemistry at the surface of diamond

**Figure 5.**
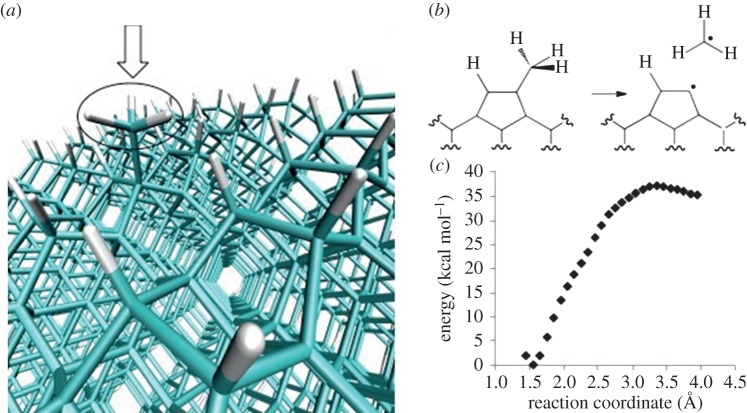
(*a*) The atomistic MD set-up upon which we carried out BXD sampling to determine PMFs for dissociation of the surface −CH_3_ group, which is highlighted by the arrow; (*b*) a schema for a simple C−C bond dissociation etching mechanism, creating a nascent methyl group; (*c*) the 1000 K PMF for methyl dissociation from a diamond surface, calculated using BXD. (Online version in colour.)

BXD is also being applied to better understand the elementary chemical mechanisms that guide diamond etching—a process wherein solid-state diamond is converted to its gas-phase atomic constituents [37]. In particular, BXD is being used to understand the kinetics and thermodynamics for dissociation of methyl radicals from diamond surfaces, according to the scheme in figure 5*a*,*b*. Spontaneous thermal dissociation of C−C bonds at the surface of diamond has been suggested as a possible etching mechanism. Preliminary PMFs along the C–CH_3_ stretching coordinate using BXD in conjunction with a molecular dynamics simulation set-up shown in figure 5*c* (at 1000 K) suggest free energy barriers to methyl dissociation which are on the order of 38–46 kcal mol^−1^, depending on temperature. The magnitude of these free energy barriers is consistent with etching of diamond surfaces occurring as a result of spontaneous loss of methyl radicals.

### (d) Solution phase dynamics studies

Another application where BXD has proved invaluable is in the area of solution phase reaction dynamics [38]. In particular, this method has permitted atomic level insights into solution phase bimolecular reaction dynamics for the following hydrogen abstraction systems: (i) CN+cyclohexane [CN+C_6_H_12_→HCN+C_6_H_11_] in dicholoromethane solvent [17,18,39], and (ii) CN+tetrahydrofuran [CN+C_4_H_8_O→HCN+C_4_H_7_O] in tetrahydrofuran solvent [40].

For these studies, the utility of BXD arises from the fact that it provides an integrated methodology for (i) efficiently mapping the solution phase PMF, and (ii) accelerating reactive events that would otherwise not be possible to simulate. To obtain PMFs for these solution phase systems, ‘boxes’ were defined in terms of the distance between the approaching CN and an H atom on the co-reactant. Figure 7*c* shows results for CN+C_6_H_12_.

PMF sampling in BXD is particularly elegant in these sorts of systems; however, the principal advantage of BXD for the aforementioned solution phase systems is its ability to provide rare event acceleration that furnishes meaningful dynamics insights. These studies were carried out by placing a BXD box boundary to the reactant side of the transition state, and ‘locking’ the trajectory so as not to go beyond this boundary towards reactants. This approach [9] guarantees that the system does not stray too far towards isolated reactants, and simultaneously accelerates transition state passage. So long as the BXD box boundary is far enough away from the transition state, then the post-transition state dynamics are decorrelated from dynamical inversion events and provide statistically meaningful dynamics results [2,9]. Conveniently, this provides us dynamical insights into how the system behaves when unperturbed by BXD box.

The high-quality rare event statistics available with the BXD technique have permitted several new insights into solution phase dynamics. One of the most interesting is the direct observation of two distinct post-reaction non-equilibrium relaxation regimes [19,39]. For example, in the case of the CN+C_6_H_12_ reaction in CH_2_Cl_2_ solvent, the first relaxation regime follows in the immediate wake of the reaction, when the nascent HCN is in close proximity to its cyclohexyl coproduct, and strong interaction between co-products facilitates rapid energy transfer and correspondingly fast HCN relaxation. The second relaxation regime occurs once HCN and cyclohexyl have diffused away from one another and into the bulk solvent. HCN relaxation is considerably slower following diffusion, owing to weaker interactions between HCN and the CH_2_Cl_2_ solvent.

**Figure 6.**
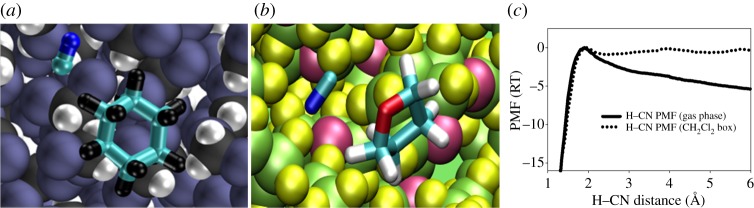
(*a*) A schematic of CN+cyclohexane in dichloromethane solvent; (*b*) CN+THF in THF solvent; (*c*) the PMF calculated for CN+cyclohexane. (Online version in colour.)

For the systems shown in figure 6, the potential energy surfaces for reaction were obtained using a multiconfigurational molecular mechanics approach, with Hamiltonian matrix elements fitted to configuration space points along the reaction path, calculated using high level CCSD(T) electronic structure theory results extrapolated to the infinite basis set limit. This approach provides a means for smoothly and accurately connecting molecular mechanics forcefields that describe both the reactant and product configuration. QM/MM approaches would have similarly allowed us to treat the reactive process, but at a considerably increased computational cost.

### (e) Free energy of unfolding by atomic force microscopy

In a number of recent experiments [32–34], the unfolding of single protein molecules with mechanical force has been investigated using the atomic force microscope. In one class of these experiments, the proteins are pulled at a constant speed (anywhere between 40 and 4000 nm s^−1^); in another class, the proteins are pulled at a constant force. The net result of such experiments is a large quantity of experimental data with detailed information on force as a function of peptide extension.

**Figure 7.**
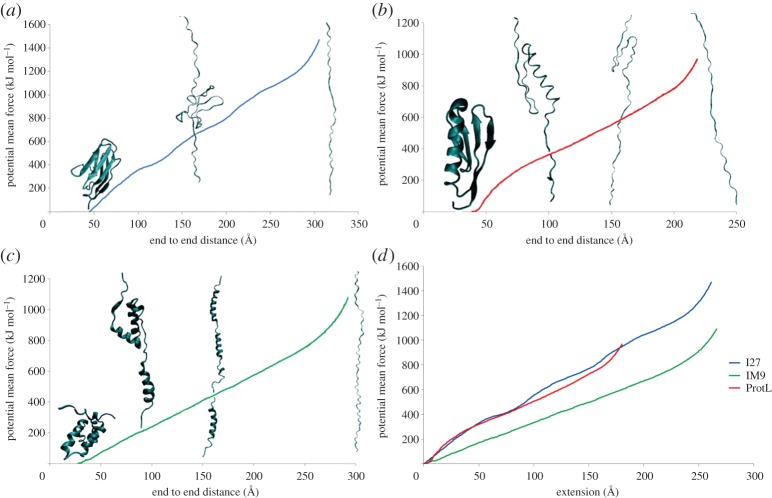
Potential of mean force profile as a function of end-to-end distance for the proteins (*a*) I27, (*b*) IM9 and (*c*) L. (*d*) A comparison of the respective PMFs. The slope of free energy is significantly smaller for protein IM9, which has only *α*-helical secondary structure. Snapshots of structures along the PMF profile are also shown. (Online version in colour.)

Because the time scale of the pulling experiment is quite long, atomistic MD simulations tend to use pulling speeds and forces which exceed those of experiment by many orders of magnitude. BXD may be easily applied to simulations of AFM due to the fact that the unfolding process is reasonably well described using a simple end-to-end distance order parameter (similar to the peptide cyclization example considered above). This allows us to investigate events that would otherwise be far too expensive to simulate. In what follows, we present the first preliminary results of BXD simulations applied to understand AFM unfolding experiments. We calculated free energies along the reaction coordinate for three proteins, all of which are shown in figure 7: IM9 is an all-alpha protein; I27 is an all-beta protein and protein L is an alpha–beta protein. Experimentally, the AFM results showed a correlation between the mechanical strength of the proteins and their secondary structure, with β-proteins requiring a much higher unfolding force than α-helical proteins.

Figure 7 shows PMF as function of end-to-end distance with results obtained using the previously discussed decorrelation algorithm [2]. The extension coordinate of proteins IM9, I27 and L were split into uniform boxes with sizes of 3, 2 and 1 Å, respectively. The average derivatives d*G*/d*x* (calculated by finite difference over an interval of 20 Å) in figure 7 are as follows: approximately 5 pN for protein IM9, approximately 170 pN for ProteinL, and approximately 170 pN for I27. These values are in broad agreement with trends reported for the experimental unfolding forces: approximately 20 pN for IM9, 185–200 pN for I27 and 100–200 pN for ProteinL (depending on the pulling speed) [32–34].

These results are preliminary, but nevertheless show qualitative agreement with the experimental trend. In future work, we will perform an extensive range of checks in an attempt to unravel the unfolding pathways, and determine how the dynamical pathways provided by BXD map into simulations compare with simulations carried out with an applied force. We will also perform kinetic studies of the extension dynamics and obtain time-dependent forces and distances under various experimental conditions—e.g. varying the pulling speed and the direction of pulling, which should permit a more direct comparison with experiment. For example, if a protein is pulled by the force *F*, then it is possible to calculate a force-modified PMF by adding the term −*Fx* to the PMF, and correcting the corresponding the box-to-box rate constants by the factor 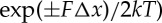, where Δ*x* is the box size. Initial kinetic simulations indicate that end-to-end distance starts increasing, and a protein begins unfolding when the force becomes comparable to the slope of the free energy plot.

## 3. Conclusion and future developments

In this paper, we presented a brief summary of the BXD method which allows the simultaneous calculation of thermodynamics (free energies along reaction coordinate) and kinetics of complicated molecular systems. We reviewed previous applications of BXD across a range of different chemical systems, such as peptide cyclization and solution phase bimolecular reaction dynamics. We also report first results obtained from simulating desorption of ions from the surface of a self-assembled monolayer, the kinetics of diamond etching, and preliminary results of simulations of protein unfolding in AFM. Atomistic simulations of these processes by standard MD are not possible, but BXD yields accurate and physically meaningful results. BXD is a simple method which is easy to implement, and which can accurately calculate free energies along a reaction coordinate or some appropriately partitioned region of molecular phase space. BXD allows one to recast the complicated atomistic dynamic evolution a given system as a set of kinetic processes occurring within different regions of configuration space. Although BXD is a fully atomistic technique, it is capable of describing dynamics many orders of magnitude longer than typical atomistic dynamics time scales. The systems outlined in this paper demonstrate how BXD has furnished considerable microscopic insights into the atomistic processes and mechanisms at work in a range of condensed phase and heterogeneous systems.

A number of BXD projects are currently underway in our groups, including the dynamics of diamond etching, additional solution phase reaction systems, the dynamics of AFM unfolding, the dynamics of pocket opening in biomedically important proteins (where access to the docking site is blocked by structural elements of the protein) and the folding dynamics of a range of small proteins. In a number of these systems, we obtain free energy/PMF profiles as well as the long-time kinetics/dynamics, furnishing considerable microscopic insights into a range of problems. The applications reported and reviewed in this paper have used relatively simple reaction coordinates, but we are presently exploring more sophisticated reaction coordinates as well as multidimensional extensions of BXD.
